# Extracting biomedical events from pairs of text entities

**DOI:** 10.1186/1471-2105-16-S10-S8

**Published:** 2015-07-13

**Authors:** Xiao Liu, Antoine Bordes, Yves Grandvalet

**Affiliations:** 1 Sorbonne universités, Université de technologie de Compiègne, CNRS, Heudiasyc UMR 7253, 57 avenue de Landshut, CS 60319, 60203 Compiègne cedex, France

**Keywords:** Information Extraction, Natural Language Processing, Machine Learning

## Abstract

**Background:**

Huge amounts of electronic biomedical documents, such as molecular biology reports or genomic papers are generated daily. Nowadays, these documents are mainly available in the form of unstructured free texts, which require heavy processing for their registration into organized databases. This organization is instrumental for information retrieval, enabling to answer the advanced queries of researchers and practitioners in biology, medicine, and related fields. Hence, the massive data flow calls for efficient automatic methods of text-mining that extract high-level information, such as biomedical events, from biomedical text. The usual computational tools of Natural Language Processing cannot be readily applied to extract these biomedical events, due to the peculiarities of the domain. Indeed, biomedical documents contain highly domain-specific jargon and syntax. These documents also describe distinctive dependencies, making text-mining in molecular biology a specific discipline.

**Results:**

We address biomedical event extraction as the classification of pairs of text entities into the classes corresponding to event types. The candidate pairs of text entities are recursively provided to a multiclass classifier relying on Support Vector Machines. This recursive process extracts events involving other events as arguments. Compared to joint models based on Markov Random Fields, our model simplifies inference and hence requires shorter training and prediction times along with lower memory capacity. Compared to usual pipeline approaches, our model passes over a complex intermediate problem, while making a more extensive usage of sophisticated joint features between text entities. Our method focuses on the core event extraction of the Genia task of BioNLP challenges yielding the best result reported so far on the 2013 edition.

## Background

Huge amounts of electronic biomedical documents are generated daily; for example, over one million published papers have been collected on Medline in 2013. Automatically organizing their content in dedicated databases enables advanced search and eases information retrieval for researchers in biology, medicine or other related fields. Nowadays, these data sources are mostly in the form of unstructured free text, which is complex to incorporate into databases. Hence, many text-mining research initiatives are organized around the issue of automatically extracting information from biomedical text. Efforts specifically dedicated to biomedical text are necessary because standard natural language processing tools cannot be readily applied to extract biomedical events since such texts, articles or reports involve domain-specific jargon, shorthands, such as "IL-1, -2, -3" for the *Interleukin *proteins [1].

**Figure 1:**
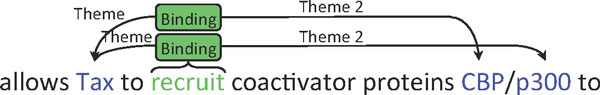
**Sentence and associated events**. Excerpt from the BioNLP 2013 Genia task.

This paper tackles the problem of event extraction from biomedical documents. Building on previous advances in named entity recognition (for detecting gene or protein mentions for instance), this task consists in associating to these entities the related events expressed in the text. Such events are of multiple types and involve at least one text entity as *argument *and another as *trigger; *they can be quite complex since some events have several arguments, and recursive in the sense that arguments can themselves be events. An example of an event is given in Figure 1.

Biomedical event extraction is attracting more and more attention, especially thanks to the organization of recurrent dedicated BioNLP challenges [2-4]. We propose here RUPEE (for RecUrsive Pairwise Event Extractor), a new approach which relies on a single multi-class classifier for recursively detecting events from (*trigger, argument*) pairs. Compared to standard pipeline approaches based on sequences of classifiers [5,6], we avoid the intermediate problem of associating isolated triggers to event types, relying on a tricky multi-label classification problem. Instead, we directly extract compounds of events in the form of (*trigger, argument*) pairs, simply relying on a multi-class problem, whereby (*trigger, argument*) pairs are associated to event types. Considering pairs of text entities also allows us to characterize examples by sophisticated joint features, such as shortest path in the dependency parse tree, and hence to detect triggers more accurately than with pipeline models. Pipeline models eliminate early text entities that act unusually as triggers; extracting the (*trigger, argument*) pairs allows to retrieve these triggers provided they are recognized to be strongly related to an *argument*. Our performance analysis of the Results section explicitely tests the added value of the pairwise approach. Besides, compared to Markov random fields [7], RUPEE is a discriminant model that does not represent the full joint distribution of words and events. We thus have a simpler inference process, which results into drastically reduced training times: roughly 15 times faster for processing about 800 training documents (on the same machine).

In short, we propose in this work a *happy medium *between pipeline and joint models. Our approach builds on our previous proposal [8], where we detected triggers directly from (*trigger, argument*) pairs. Here, we upgrade our scheme by adding a recursive classification process that considerably improves the detection of complex events. As shown in the Results section, RUPEE reaches the best performance reported so far on the BioNLP 2013 Genia task and the second best performance on the BioNLP 2011 Genia task, with a reduced training duration compared to the previously released models.

### Biomedical event extraction

Biomedical event extraction aims at extracting *event formulas *from sentences, defined as sequences of *tokens *(words, numbers, or symbols). We first introduce the main concept of the task and present the data provided by the BioNLP challenges.

#### Definitions

Terminology regarding biomedical events, triggers, etc. varies from one task or data set to another; in the following, we use the definitions used by the Genia (GE) task 1 of the BioNLP challenges. An event is defined as a formula constituted of two kinds of elements: an event *trigger *and one or several *arguments*. The event trigger is a *text entity *to be extracted, whereas arguments can be proteins, genes or other events. We define a text entity as a character string, which can be a part of word or several consecutive words. All the *entity *statements mentioned in this paper refer to text entity. In the data settings of the GE task, gene/protein mentions are already annotated in the text. Nine types of events, listed in Table 1, are defined in the BioNLP GE task. These nine types may be merged into three broader categories: first, the single *theme *argument events (SVT); then the Binding events (BIN), which may take up to two theme arguments; and finally, regulation events (REG), which may take up to two arguments, a theme and an optional *cause*. REG events are possibly recursive because their arguments may be either proteins or events. Thirteen types of events were defined in the BioNLP 2013 challenge, but we only dealt with the nine types originally defined in the previous challenges, because the newly defined types were represented by too few examples for proper training.

**Table 1:** Classes and types of events with their arguments (P stands for *Protein*, E for *Event*).

*Class*	*Type*	*Principal arg*	*Optional arg*
	Gene expression	theme (P)	
S	Transcription	theme (P)	
V	Protein catabolism	theme (P)	
T	Phosphorylation	theme (P)	
Localization	theme (P)		

B			
I	Binding	theme (P)	theme_2 (P)
N			

R	Regulation	theme (P/E)	cause (P/E)
E	Positive regulation	theme (P/E)	cause (P/E)
G	Negative regulation	theme (P/E)	cause (P/E)

Figure 1 illustrates biomedical event extraction in the GE task framework: given three proteins *"Tax*", *"CBP*" and *"p300*", one must detect two events of the Binding category, both associated to the *"recruit*" trigger: *("recruit*", theme: "*Tax*", theme_2:"CBP") and (*"recruit*", theme: "*Tax*", theme_2: "p300"). A key part of the task is to detect the trigger entities among the candidate sequences of tokens.

#### Data

Three BioNLP GE tasks have been organized: in 2009, 2011, and 2013. Each time, three separate data sets were produced for training, development, and test purposes. These data sets were created from extracts of articles from PubMed; training and development sets contain fully annotated texts, with events (triggers and arguments) and their types, whereas test sets contain texts annotated with protein mentions alone. Extracts from either titles, abstracts or full articles have been used, following the repartition reported in Table 2. We conducted our experiments using the test data from 2011 and 2013 editions. When evaluating on data from 2011, we used the corresponding training and development sets; when evaluating on data from 2013, we gathered the training and development sets from 2011 and 2013.

**Table 2:** Statistics of corpora of BioNLP GE tasks.

	Training		Development		Test	
**Year**	**Tit./Abs**.	**Full**	**Tit./Abs**.	**Full**	**Tit./Abs**.	**Full**

2009	800	0	150	0	260	0

2011	800	108	150	109	260	87

2013	0	222	0	249	0	305

**Figure 2:**
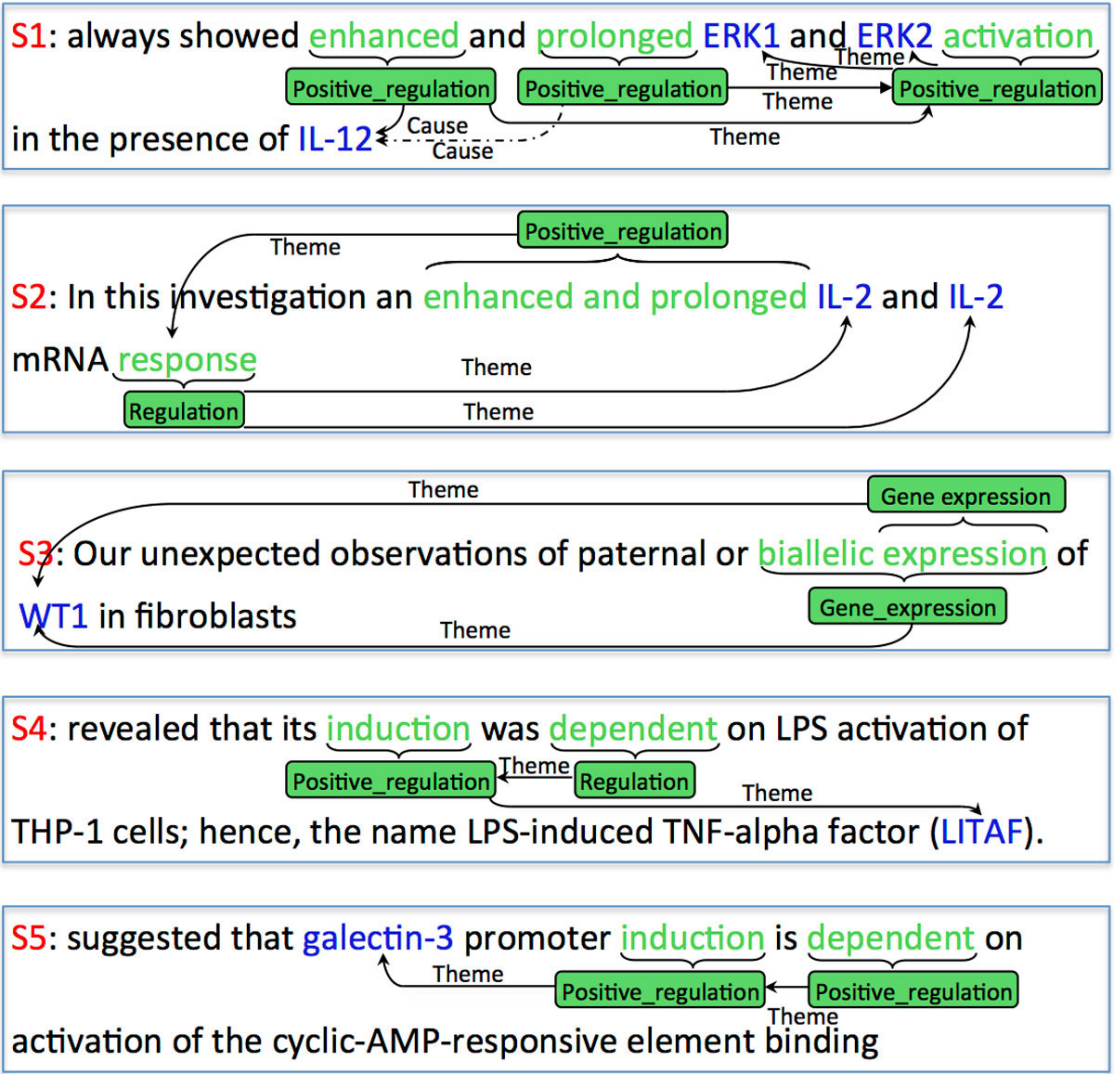
**Examples of ambiguous annotations**.

Working with texts extracted from titles, abstracts, or full papers has consequences, as it has been previously shown that the distribution of events varies according to the position in articles [9]. More problematic, we remarked that annotations are also affected by the position: for instance, the trigger "*overexpress*" is always labeled **Gene_expression** and **Positive_regulation** in abstracts and titles, whereas it is exclusively labeled by only one of those types in full papers. Figure 2 illustrates other difficulties that arise due to the manual annotation of data: in S1, "*enhanced and prolonged*" is split as two triggers, whereas it is labeled as a single trigger in S2; in S3, two **Gene_expression** events are related to the same protein, with nested triggers "*expression*" and "*biallelic expression*"; in S4, the trigger "*dependent*" is labeled as Regulation, whereas in S5 it is labeled as **Positive_regulation** in a similar context, which does not give any hint on the positiveness of the regulation. Clearly, there are some fluctuations in the annotations, which may represent several legitimate ground truths, but they are hardly reproducible by a classification algorithm. These fluctuations are likely to affect the evaluation in a fair way regarding the comparison of classifiers, but in an adverse way regarding sheer performance. The annotation of REG events is particularly subtle and some of the confusions made by automatic systems may reflect the actual uncertainty about ground truth that arises from the ambiguity of some sentences. Even if the consistency of gold annotations has not been assessed in the Genia task, we may assume that it is omparable to the one of the the ID (Infectious Diseases) task, which is very similar to the Genia task. This consistency was found to be below 75% in a previous study [10].

## Methods

We describe now our model RUPEE; it directly extracts pairwise interactions between entities, thereby contrasting with the usual pipeline approaches, which require detecting triggers as an intermediate problem. RUPEE proceeds in two steps:

**Main (trigger, theme) pair extraction **that detects the triggers with one of their arguments;

**Post-processing **that adds extra arguments to BIN and REG events.

### Recursive pairwise model

This section first details the first step, which is the main innovative part of our system.

#### Direct extraction of simple events

We process entities differently depending on whether they are marked as proteins in the annotation or not; the latter are termed *candidate *entities. In a given sentence *S*, we denote *C_S _*= {*c_i_*}*_i _*the set of candidate entities, which is built from the sentence tokens, and *A*_S _= {*a_j_*}*_j _*the set of candidate arguments (that is, the proteins identified by a named-entity recognizer beforehand). The set of event types (augmented by None) is denoted Y.

The first steps of a pipeline model consist in predicting whether candidate entities *c_i _*∈ *C_S _*are triggers or not and then, whether arguments *a_j _*∈ *A_S _*can participate to a subset of events from *Y*. Instead, our pairwise model directly addresses the problem of classifying the (candidate, argument) pairs *p_ij _*= (*c_i_, a_j_*) as events of type from *Y*. This classification is based on Support Vector Machines (SVMs), where the multi-class problem is broken down in a series of one-vs-rest binary problems, one for each event type. The final decision associated to each pair *p_ij _*is simply taken as the event (including None) whose score is maximal. As a result, classifying a pair *P_ij _*as not-None jointly detects the event trigger *c_i _*and its argument *a_j_*.

#### Recursive extraction of complex events

For simple SVT and BIN events, the set *A*_S _of possible arguments is restricted to proteins, but the events of class REG may have other events as arguments, thus *A_S _*has to be enriched. Considering all possible events would be intractable, so that the set of possible arguments is updated dynamically in the process of extracting events. As these new argument can only be assigned to some specific event types (that is, REG events), in practice it is simpler to update the set of pairs that remain to be assessed.

Assume that an event has been actually predicted, that is, that *p_αβ _*= (*c_α_, a_β_*) has been classified as ${\hat{y}}_{\alpha \beta }\neq \text{None}$; the predicted event is denoted ${\hat{e}}_{\alpha \beta }=\left(c_{\alpha },a_{\beta },{\hat{y}}_{\alpha \beta }\right)$. We then create all pairs with it as argument, {(*c_i_, c_α_*)|*c_i _*∈ *C_S_*}, and add them to *P_S_*, so as to detect recursive events. We assume that recursive events constitute a directed acyclic graph, where the ancestor of a candidate entity cannot be used as its argument. The dynamic updating process is thus constrained to prevent the creation of cycles.

**Algorithm 1 **Extracting events with RUPEE

**input **sentence *S*, candidate entities *C_s _= *{*c_i_*}*_i_*, labeled proteins *A_s _= *{*a_j_*}_j _and binary classifiers *f_k _*for each event type *k*

1: **initialize **candidate pairs

  *P_s _*= {(*c_i_, a_j_*), *c_i _*∈*C_s_, a_j _*∈ *A_s_*}

2: **initialize **extracted events *ε_s_=∅*

3: **for ***p_αβ_* ∈ *P_s _***do**

4:   score $s_{\alpha \beta }^{k}=f_{k}\left(p_{\alpha \beta }\right)$ for each event type *k*

5:   store ${\hat{s}}_{\alpha \beta }={\text{max}}_{k}s_{\alpha \beta }^{k}$ and ${\hat{y}}_{\alpha \beta }=\text{arg}{\text{ max}}_{k}s_{\alpha \beta }^{k}$

6: **end for**

7: **while ***P_S _*≠ *∅n ***do**

8:   select the pair $p_{\alpha \beta }\in P_{S}$ such that $p_{\alpha \beta }=\text{arg}{\text{ max}}_{\alpha \beta }{\hat{s}}_{\alpha \beta }$

9:   update $P_{S}\leftarrow P_{S}-\left\{p_{\alpha \beta }\right\}$

10:   **if **${\hat{y}}_{\alpha \beta }\neq \text{None}$**then**

11:     create event ${\hat{e}}_{\alpha \beta }=\left(c_{\alpha },a_{\beta },{\hat{y}}_{\alpha \beta }\right)$

12:     update $E_{S}\leftarrow E_{S}\cup \left\{{\hat{e}}_{\alpha \beta }\right\}$

13:     update *P_S _*← *P_S _*∪ {(*c_i_, c_α_*)|*c_i _*∈ *C_s_*}

14:     censor pairs in *P_S _*to avoid cycles

15:     compute *$\hat{s}$*and *$\hat{y}$*for the new {(*c_i_, c_α_*)} pairs

16:   **end if**

17: **end while**

18: return extracted events *Ε_S_*

Algorithm 1 summaries our event extraction algorithm RUPEE. For all events with a single argument, predicting y variables directly responds to the event extraction problem. When appropriate, additional optional arguments are added after all pairwise events have been extracted, by the post-processing. Working on pairs allows us to take into account interactions, in particular through dedicated features describing the links between the trigger and its argument.

#### Fitting the pairwise model

The prediction process described above relies on a multi-class classifier. We stress again that, since pairs are assigned to a single class, there is no need to address the more difficult multi-label problem encountered in standard pipeline approaches. An entity may still be assigned to several events, possibly of different types, through the allocation of labels to several pairs comprising this entity. We now report some important details on the learning process of RUPEE.

For each event type, a series of binary linear SVMs is fitted to the available training data, using the Scikit-learn implementation [11]. As events are rare, each binary classification problem is highly imbalanced. We thus use different losses for positive and negative examples [12,13], resulting in two hyper-parameters C^+^/C^- ^that are selected by cross-validation from a set of candidate values (C^+ ^∈ {0.001, 0.01, 0.1,1,10,100} and C^- ^∈ {0.001,0.01, 0.05,0.1,1,10}). The selected C^+^/C^-^pair maximizes the cross-validated F-score of the corresponding event type (taken in isolation).

For the SVT and BIN events, the training sets are all composed of the possible (candidate, argument) pairs $Ps=\left\{p_{ij}=\left(c_{i},a_{j}\right)|c_{i}\in Cs,a_{j}\in As\right\}$ readily extracted from all training sentences, and they only differ in the definition of the positive and negative class, according to the true label associated to each pair.

Creating the training sets for REG events is more complicated: since they can take events as arguments, new pairs are added to *P_S _*by considering all the events already detected, as sketched in Algorithm 1. Hence, the sets of training examples are not deterministically known before training, but depend on predictions of all other classifiers. Training directly on them requires to use either online algorithms or complex search-based structured prediction procedures as in [14]. In this paper, we prefer to use instead the true labels $y_{\alpha \beta }$ during the training phase of REG and None classifiers: the training sets are then the enriched sets of possible (candidate, argument) pairs $Ps=\left\{p_{ij}=\left(c_{i},a_{j}\right)|c_{i}\in C_{s},a_{j}\in A_{s}\right\}\cup \{p_{i\alpha }=\left(c_{i},c_{\alpha }\right)|c_{i}\in$$Cs,\;\exists \beta :y_{\alpha \beta }\neq \text{None\}}$. This allows to know all training examples beforehand and hence to use standard batch SVM algorithms. The drawback is that, since extracted events in test are imperfect, this creates a divergence between training and testing scenarios, which can lead to degraded performance. However, as our experiments show, this effect is marginal compared to the advantages of using fast reliable batch training algorithms for SVMs.

The final decision rule simply consists in predicting the class corresponding to the highest SVM score. This simple scheme could be improved, either by using multi-class classifiers or by using more refined combinations optimizing a global criterion as in [8]. Though this route deserves to be thoroughly tested, we conjecture that only marginal gains should be expected since, as the confusion matrix of Table 3 shows, the vast majority of errors are due to the detection of an event when there is none or to the absence of detection of an existing event: when an event is rightly detected, its correct type is predominantly predicted.

**Table 3:** Confusion matrix for RUPEE on the BioNLP 2013 GE task, computed by cross-validation on the training and development sets.

*Predicted / True*	None	Gene exp	Trans	Pro cat	Phosp	Local	Bind	Regu	Pos reg	Neg reg	
None	223460	404	163	27	42	60	296	390	799	397	226038

Gene expression	440	2741	13	0	0	17	2	1	43	5	3262
Transcription	186	16	565	0	0	0	0	4	15	0	786
Protein catabolism	30	0	0	150	0	0	0	0	1	0	181
Phosphorylation	76	0	0	0	413	0	0	0	0	0	489
Localization	114	20	0	0	0	398	4	0	1	2	539

Binding	507	0	0	0	0	1	1470	2	0	1	1981

Regulation	453	0	0	0	0	0	1	813	33	4	1304
Positive regulation	1245	42	10	0	0	0	2	67	2456	7	3829
Negative regulation	555	7	2	1	1	0	0	46	11	1176	1799
	227066	3230	753	178	456	476	1775	1323	3359	1592	

#### Computational considerations

The pairwise structure leads to a simple inference procedure, with a slight increase in computational complexity compared to pipeline models. We denote *m *= card (*C_S_*), the number of candidate entities, *n *= card (*A_S_*), the number of annotated proteins and m' the number of detected triggers. The complexity of a pipeline model is *O *(*m*'(*n *+ *m*')), whereas that of RUPEE is *O *(*m*(*n *+ *m*')). Our complexity is bigger than the pipeline model but cheaper than joint models such as the one introduced in [7], whose complexity is *O*(*m*(*n*^2 ^+ *m*)).

### Post-Processing

We now describe the post-processing carried out once the (trigger, theme) pairs are detected and labeled as events. The goal is to look whether secondary arguments should be added to these extracted events.

#### Binding theme fusion

This step attempts to merge several pairs labeled as Binding to create multiple arguments events. We take the set of extracted Binding events {(*c_α_, a_β_*)} that share the same trigger *c_α_*, and all combinations {(*c_α_, a_β , _a_γ_*)| γ ≠ β} are classified by a binary SVM. Once a combination (*c_α_, a_β , _a_γ_*) is predicted as a correct merge, it is added to predicted events while both pairs (*c_α_, a_β_*) and (*c_α_, a_γ_*) are removed.

#### Regulation-Cause assignment

This step looks for optional cause arguments that may be added to the extracted REG events. Given an extracted event (*c*_α _, *a*_β_) and a candidate argument set *A_S _*= {*a*_γ_} containing all the proteins of the sentence *S *as well as all events extracted by the classifier, all combinations {(*c_α_, a_β_, a_γ_*)|γ ≠ β} are classified by a binary SVM. Since *cause *argument could be another event, we extract them incrementally in a dynamic process alike (trigger, theme) pair extraction, also with constraints avoiding the creation of cycles.

### Features

This section details our features as well as the data preprocessing used by RUPEE.

#### Pre-Processing

Tokenization and sentence splitting have a substancial impact on the results of the dependency parsing as well as the way we handle compound words that contain protein names. We split the data in sentences using both the nltk toolkit [15], and the sentence splitting provided for the BioNLP GE task. As the best pipeline model TEES and the best joint model UCLEED use fine-grained tokenizations, we conjectured that fine-grained tokenizations could provide relevant features. Coarse tokenizations are useful to maintain some biomedical jargon that also convey essential information. Compared to using dependency parse trees based solely on coarse tokenizations, adding features from the dependency parse trees based on fine-grained tokenizations improves the total F-score of our RUPEE model by more than 2% on the BioNLP 2013 test set. Hence, two tokenizations are used for different features. The main difference between coarse and fine-grained tokenizations is that some compound words are split by handcrafted rules in fine-grained tokenization. For example, given a sentence "*inhibit NF-kappaB-dependent pro-inflammatory gene transcription*", "*NF-kappaB-dependent*" is split into two words "*NF-kappa*" and "*dependent*" in fine-grained tokenization, but kept as a single word in coarse tokenization. *Tokenization1*, provided by the organizers of the BioNLP GE task, is a coarse tokenization that is used to characterize when a candidate entity and a protein are in the same token, the parsing results provided by organizers are all based on this tokenization. *Tokenization2 *is fine grained, based on the Stanford parser [16] that is slightly modified for primary tokenization. It supplies the dependency parse, candidate entity match and most of our features. We used stems (obtained by the Snowball stemmer provided in nltk) as base forms for the tokens. In order to get the parse trees based on the fine-grained tokenization, we computed them using a phrase structure parser [17], along with the post-processing of the Stanford corenlp package [18].

#### Candidates

For each sentence *S*, the set *C_S _*is built with a gazetteer: candidate entities are recursively added by searching first the longest token sequences (from Tokenization2) from the gazetteer. For entities with several tokens, a representative *head *token is selected by a heuristic based on the dependency parse.

Three types of tokens are considered: the head token, its parent and child nodes in the dependency tree, and the tokens belonging to a neighboring window of the entity. The size *k *of the word window is a hyper-parameter of our model. Table 4 lists all features which include stems, part-of-speech (POS) tags, etc. Special care was taken to design the feature for head token since it plays an extremely important role in candidate entities. We hence employed features and heuristics to deal with compound-words, hyphens and prefixes, inspired by such tools developed in the code of the UCLEED system and based on Tokenization2. Protein names and POS in tokens are substituted by the token PROT, e.g. transforming "LPS-activated" into "PROT-activated". In the end, there is total of a 35,365 candidate features.

**Table 4:** Features used by our system. Most are based on Tokenization2 except when specified

	Features	Examples
*Candidate entity features*	Base form (stem) of the head token.	regul for tokens "regul*" (e.g. "regulation" in Figure 3).
	
	Base form of the head token without '-' or '/' before of after.	depend for token "-dependent"

	Sub-string after '-' in the head token.	dependent for token "-dependent"
	
**Figure 3:**
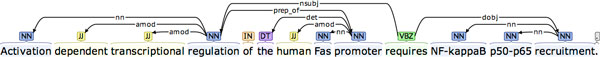
**Example Sentence with Part-Of-Speech Tags and Dependency Parse**.

	POS of the head token.	VBZ for token "requires" in Figure 3
	
	First token of the entity is after '-' or '/'.	-First for entity "-independent pathways"
	
	Last token of the entity is before '-' or '/'.	Last- for entity "phobol ester-"
	
	Head token has a special prefix: "over", "up", "down", "co"	up for "upregulation"
	
	Concat. of base form and POS of parents of the head token in dependency parse.	NSUBJ←requir/VBZ for "regulation" in Figure 3
	
	Concat. of base form and POS of children of the head token in dependency parse.	NSUBJ→regul/NN, DOBJ→recruit/NN for "requires" in Figure 3
	
	Base forms of *k *neighboring tokens around the entity.	Base forms from the 2nd previous token to the 2nd next token are PROT, promot, PROT, PROT for "requires" in Figure 3
	
	POS of k neighboring tokens around the entity.	POS from the 2nd previous token to the 2nd next token are JJ, JJ, IN, DT for "regulation" in Figure 3
	
	Neighborhood of the entity has '-' or '/'.	Features from the 2nd previous token to the 2nd next token are NONE, NONE, hyphen, hyphen for "requires" in Figure 3
	
	Sentence has "mRNA".	True if "mRNA" exists in any position of the sentence
	
	Entity is connected with another string using Tokenization1.	PROT-expression and PROT-express for token "Tax-expression"

*Argument features*	Argument is a protein.	True if the argument entity overlaps any protein
	
	POS of the head token.	NN for "NF-kappa" in Figure 3
	
	Features extracted from IntAct when the argument is a protein.	association, physical association for protein name "c-Rel"
	
	Base forms of k neighboring tokens around the argument.	Base forms from the 1st previous token to the 1st next token are requir, PROT for "NF-kappa" in Figure 3
	
	POS of k neighboring tokens around the argument.	POS from the 1st previous token to the 1st next token are VBZ, IN for "NF-kappa" in Figure 3
	
	Concat. of base form and POS of parents of the head token in dependency parse.	NSUBJ←requir/VBZ for "regulation" in Figure 3

*Joint features*	Token sequence between candidate and argument has proteins.	[PROT] ... PROT ... [trigger] and [PROT] ... PROT ... [recruit] **for ("NF-kappaB", "recruitment") in **Figure 3
	
**Figure 4:**
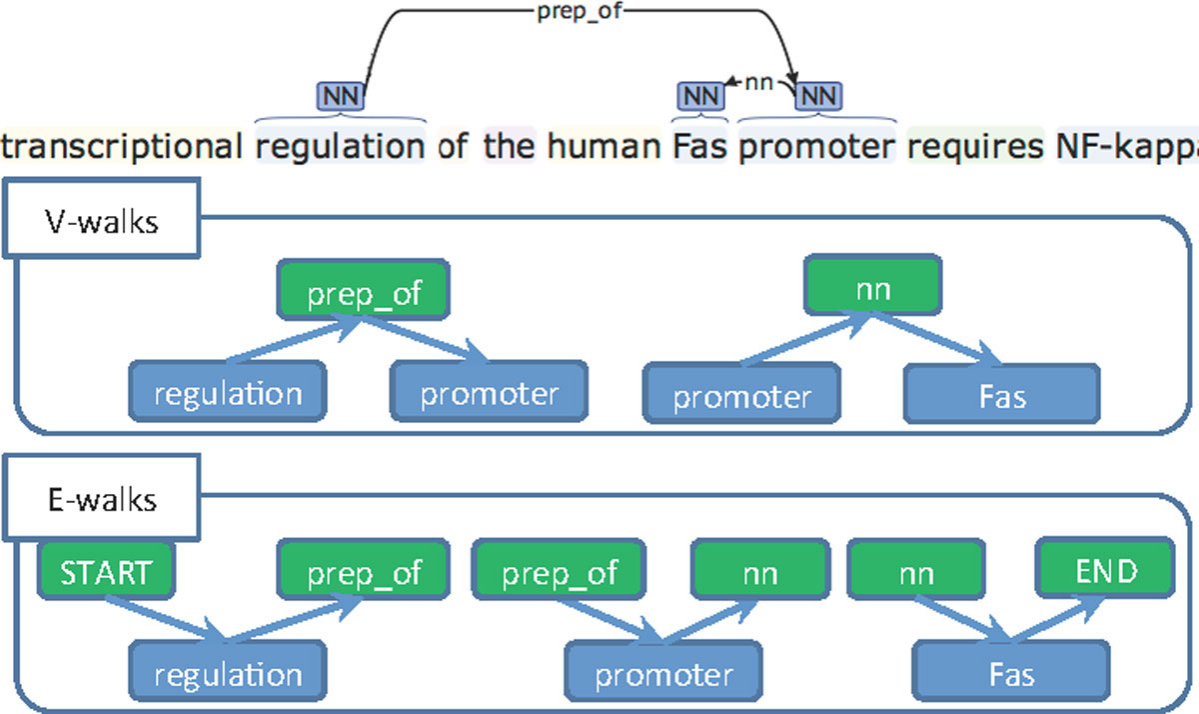
**E-walks and V-walks**. Examples of encodings of the dependency parse tree.

	V-walk features between candidate and argument with base forms.	regul $\underset{\leftarrow }{\text{PREP\_OF}}$promot, promot $\underset{\to }{\text{NN}}$ PROT **for example in **Figure 4
	
	E-walk features between candidate and argument with base forms.	$\underset{\to }{\text{START}}$ regul $\underset{\to }{\text{PREP\_OF}}$, $\underset{\to }{\text{PREP\_OF}}$, prompt $\underset{\to }{\text{NN}}$$\underset{\to }{\text{NN}}$ PROT $\underset{\to }{\text{END}}$**for example in **Figure 4
	
	V-walk features between candidate and argument with POS.	NN, $\underset{\leftarrow }{\text{PREP\_OF}}$ NN, NN, $\underset{\to }{\text{NN}}$ PROT **for example in **Figure 4
	
	E-walk features between candidate and argument with POS.	$\underset{\to }{\text{START}}$ NN $\underset{\to }{\text{PREP\_OF}}$ NN $\underset{\to }{\text{NN}}$, $\underset{\to }{\text{NN}}$ PROT $\underset{\to }{\text{END}}$**for example in **Figure 4
	
	Candidate and the argument share a token using Tokenization1.	ARG-express **for "Tax" and "expression" in "Tax-expression"**

Most are based on Tokenization2 except when specified.

#### Arguments

Table 4 also lists the argument features, which are a subset of those for candidate entities. Most head word features are removed, but base forms and POS of the neighboring tokens and of the parent node in the dependency tree are still included. Assigning label from SVT or BIN event classes to a (*c_i_, e_αβ_*) pair should never occur, because only regulation events could have another event as argument. Therefore, we add a feature that indicates whether the argument is a protein or a trigger entity. Proteins are also described using features extracted from the Uniprot knowledge base [19]. We first queried the knowledge base using the protein name strings marked in annotation to create a map from protein names to protein IDs, then retrieve the protein interaction information from the IntAct knowledge base [20]. Since the protein name strings were usually an imperfect match, we collected the top 5 IDs for each protein name string and supposed that protein name strings that share the same top 5 IDs have identical interaction knowledge. There is total of 4,349 argument features.

#### Pairwise relations

Our pairwise approach is able to take advantage of the joint features listed in Table 4, which code interactions between candidate triggers and arguments. Hence, we have a feature indicating if both elements of a pair belong to the same token (based on Tokenization1).

But the most important joint features come from the shortest path linking candidates and arguments in the dependency parse tree of the sentence. Incorporating such dependency information into the pairwise model relies on the process encoding the path into feature vectors. Many formatting methods have been proposed in previous works. Following [21], our system uses a combination of *E-walks *(edge walks) which encode the path into (*dep-tag, token, dep-tag*) triplets; and *V-walks *(vertex walks), which encode the path into (*token, dep-tag, token*) triplets. In these triplets, tokens are described by stem and POS tags, and *dep-tags *are the dependency labels. Figure 4 illustrates this formatting: from the dependency parse given on top, three *V-walk *and two *E-walk *features are defined. These are inserted in the feature vector using a bag-of-words process, thus losing any relative ordering information. These imperfect representations lose a lot of information and can even add noise, especially when the path is long. Therefore, we applied heuristics from the UCLEED system to remove some uninformative edges from the dependency parse. Moreover, dependency parse features are added only for pairs for which the (candidate, argument) path length is below a threshold whose value is a hyper-parameter. There is a total of 176,106 pairwise features.

### Related work

Besides rule-based approaches, such as NCBI [22] or BioSEM [23], and pattern-based approaches such as NICTA [24], current approaches heavily rely on machine learning. These statistical approaches fall into two main categories: pipeline incremental models and global joint methods. Pipeline approaches [25-27] are the simplest way to tackle the problem of event extraction. A sequence of specific classifiers is run on the text to successively

(P_1_) detect event triggers,

(P_2_) assign arguments to triggers,

(P_3_) detect triggers whose arguments can be events,

(P_4_) assign arguments to these latter triggers.

Steps (P_3_) and (P_4_) can be ran multiple times. Such systems are relatively easy to set up and experienced many successes: the TEES system [28,29,5] won the BioNLP GE task in 2009 and ranked *2^nd ^*in 2013, whereas the EVEX system won in 2013 [30,6]. However, all these methods suffer from error cascading. Besides, prediction must be formalized as a multi-label classification problem because some words can participate in the definition of several events of different types. Detecting triggers in isolation of their arguments in steps (P_1_) and (P_3_) are ill-posed intermediate problems, since the notion of trigger is intrinsically tied to its argument. The latter brings contextual information that is indisputably relevant for detection. Besides, rich features coding for (trigger, argument) pairs [21] are used by pipeline models only for assigning arguments (that is, edge detection step in TEES and TEES 2.1), whereas they could be useful for trigger detection as well.

Global joint approaches [31,16,22,24,23] aim at solving the event extraction task at once, so as to resolve the drawbacks of pipeline models. In [16], event annotations are converted into pseudo-syntactic representations and the task is solved as a syntactic extraction problem by traditional statistical parsing methods. In [31,7,33], some models are proposed based on the maximization of a global score taking into account the annotations of nodes and edges in a graph representing each sentence. This maximization problem is formalized as an integer linear program with consistency constraints, and solved via dual decomposition. Such joint models perform very well (winner of the BioNLP 2011 GE task), but suffer from high computing costs, as all possible combinations of words are considered as potential events. In the following, we show that RUPEE is able to reach slightly better accuracies than joint models while being computationally much cheaper. A method based on the search-based structured prediction paradigm [14] has already been proposed as an intermediate step between joint and pipeline approaches, by turning the structured prediction problem into a sequence of multiclass classification tasks. Our experiments demonstrate that, despite being conceptually simpler, our recursive pairwise model can outperform it as well.

## Results

In this section, we demonstrate the performance of RUPEE in the framework of the GE tasks of the BioNLP challenges. More precisely, we use the annotated data collected for these tasks and report the results returned by the evaluation servers on the test sets of the 2013 GE task, and also of the 2011 edition so as to compare to joint methods.

To assess the efficiency of our modeling choices, we also implemented a pipeline counterpart system, following the structure of the TEES approach [28,29,5] but using our feature set, together with a pre-processing and a post-processing that best match our system. This pipeline system comprises four steps:

**Trigger classification**, which assigns event types from Y to candidate entities *c_i _*∈*C_S _*using a multi-class SVM classifier;

**Edge detection**, which identifies the edges between extracted triggers and proteins and between REG triggers and all the triggers; labels from *Y_edge _*= {*theme, cause, None*} are assigned to those pairs;

**Binding theme fusion**, which merges several pairs labeled as *Binding *to create multiple arguments events, following the post-processing used for our algorithm and described in the Methods section;

**Regulation-cause assignment**, where two predicted pairs (*c_i_*, theme: *a_β_*), (*c_i_, cause *: *a_γ_*) may be merged into a single (*c_i_, theme *: *a_β_, cause *: *a_γ_*).

### Genia shared task 2013

For the BioNLP 2013 GE task, the hyper-parameters of RUPEE have been optimized on the GE task development set (except for the regularization parameters of the SVMs, which are selected by cross-validation), after training on the corresponding training sets: token window size is 2 for candidate entities and 1 for arguments, the threshold for dependency path is 4. Using these hyper-parameter values, the final model submitted for test evaluation on the GE task server has been trained on all documents from training and development sets of BioNLP 2011 and 2013 GE tasks. Detailed descriptions of the BioNLP 2011 and 2013 GE data are respectively given in [3] and [4].

Table 5 lists the detailed test F-scores, as returned by the official challenge test server (using the default *approximate span & recursive matching *evaluation setting). We compare our model RUPEE to the winner of the challenge, EVEX [6], and of the best runner-up, TEES 2.1 [5], which are both pipeline approaches.

**Table 5:** F-scores on the test set of the BioNLP 2013 GE task.

*Event Type or Class*	TEES 2.1	EVEX	Pipeline counterpart	RUPEE
Gene expression	82.7	82.7	83.9	**85.1**
Transcription	55.0	55.0	61.7	**62.8**
Protein catabol	56.3	56.3	66.7	**68.8**
Phosphorylation	72.6	71.5	**81.8**	**81.8**
Localization	**63.3**	60.7	56.9	57.7
SVT TOTAL	74.9	74.5	79.0	**79.6**

BIN TOTAL	**43.3**	42.9	41.6	42.4

Regulation	23.0	23.4	23.1	**31.8**
Positive regul	38.7	39.2	36.5	**46.3**
Negative regul	43.7	**43.9**	38.1	43.6
REG TOTAL	38.1	38.4	35.1	**43.2**

ALL TOTAL	50.7	51.0	50.8	**54.4**

RUPEE is slightly below TEES 2.1 on BIN events, but overall, it outperforms all competitors significantly (by more than 3%), with a wide margin on REG events. Note that, since the test set is blinded, it is difficult to estimate standard errors properly. However, crude estimates of standard errors derived from the numbers returned by the evaluation server (namely, number of positive, true positive, and estimated examples) are about 1%. We thus expect lower differences to be significant, since there should be some overlap between the mistakes of the different methods.

The pipeline counterpart of RUPEE has an overall performance similar to EVEX and TEES 2.1, while being better for SVT and worse for BIN and REG events. These disparities are due to the differences in features and in processing details. The benefits of the pairwise structure and the recursive process are demonstrated by the considerable improvement upon the pipeline counterpart of RUPEE (using the same features, pre- and post-processing). In particular, the recursive prediction process run on REG events brings about a very substantial improvement (more than 8%).

### Genia shared task 2011

The best performing methods on the BioNLP 2013 GE task were pipeline approaches, but the joint models that were performing better in the previous challenge were not competing in 2013. As these joint models are quite tricky to train, we compare RUPEE with joint models on the BioNLP 2011 GE task, where trustworthy performances have been publicly released. We train our model using the training and development sets available at the time of the challenge and we then get an evaluation on the same test data using the official test server maintained online by BioNLP organizers. Table 6 lists the results of RUPEE, its pipeline counterpart, and those of UCLEED [7] and TEES [34], which are respectively the best performing joint model and best pipeline on this task. We also added SEARN [14], which is a hybrid between them. The results for FAUST, UCLEED, TEES and SEARN models are reproduced from [35,14].

**Table 6:** F-scores on the test set of the BioNLP 2011 GE task.

*Event Class*	FAUST	UCLEED	SEARN	TEES	Pipeline counterpart	RUPEE
SVT	73.9	73.5	71.8	72.1	71.8	**74.0**
BIN	48.5	48.8	45.8	43.4	40.0	**50.5**
REG	44.9	43.8	43.0	42.7	35.7	**45.1**

ALL	**56.0**	55.2	53.5	53.3	50.0	55.6

As for 2013 data, RUPEE achieves a higher F-score on all event classes compared to its pipeline counterpart. The benefits of the pairwise structure and the recursive process are larger here, thereby outperforming the overall F-score of TEES, which itself performs better than our pipeline counterpart. Systematic improvements on all event classes are also observed compared to the joint model UCLEED and to the search-based structured prediction approach of SEARN.

To our knowledge, RUPEE thus reaches the best overall performance reported so far on this data set for a single model. FAUST [36] achieves the best F-score on this task (56.0), but it stacks several models by using the predictions of a number of variants of the Stanford event parser [16] as input features in a modified UCLEED model.

By combining the use of the simple pair structure between triggers and arguments with a recursive prediction process, RUPEE is able to outperform pipeline models and to be at least at par with models relying on much more sophisticated structures. For this task, it is thus highly beneficial to consider pairwise interactions from beginning to end, but more complex dependencies seem not to be essential, especially since they come at a higher computational cost.

### Performance analysis

This section provides a more detailed analysis of the performances of RUPEE. We first show results indicating that our direct approach, avoiding the intermediate trigger detection problem, results in a series of specialized event detectors that are more accurate than the cascade of detectors of the pipeline counterpart. Then, we show that the different solutions that can be reached by tuning the precision-recall trade-off of our model dominate their pipeline counterpart.

The errors made by a classifier can be summarized by a confusion matrix, whose entries show which classes are confused with each others. As the test data is blinded, we computed the confusion matrix on the training and development sets, using cross-validation class assignments to get results representative of unknown test data. Table 3 shows this confusion matrix, which is computed as the sum of confusion matrices on left-out data during cross-validation. Each row corresponds to the examples of a given class, while each column corresponds to predictions into this class. None represents the "no event" class.

The most striking characteristic shown by the confusion matrix is the large number of zeros. In fact, the vast majority of errors are either due to undetected events or to false detections: once an event is rightly detected, predicting its class seems to be rather easy, and there is little confusion between event types. The strongest deviation to this general rule is for REG events, with some confusions between the different regulation types, but they incur a minor loss compared to undetected events and false detections. In fact, our approach builds a collection of specialized event detectors that by-pass the intermediate trigger detection problem of pipeline models. As shown in Tables 5 and 6, this collection of specialized detectors is more accurate than the the cascade of the pipeline counterpart.

We now compare the precision-recall trade-offs that can be reached by RUPEE and its pipeline counterpart. As shown in Table 3, most classification errors are either undetected events or false detections. We adjust the detection rate by shifting the decision threshold on the None class. As before, since the test data are blinded, these curves are computed on the development set of BioNLP 2013, for models adjusted on the BioNLP 2011 and 2013 training data.

**Figure 5:**
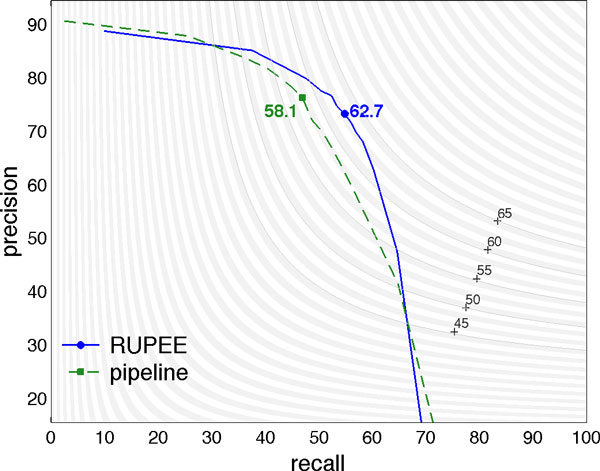
**Precision-recall curves of (trigger, theme) pairs classification with level curves of F-score in the background, computed on the BioNLP 2013 development set**.

Figure 5 displays the precision-recall curves for (trigger, theme) pair extraction, that is, before the post-processing that is common to the two approaches. The positions and the F-scores of the actual classifiers are marked in bold, and the level curves of F-scores are displayed in the background. Note that these F-scores are not necessarily maximal since the classifiers are not calibrated on the test set. The maximal values of recall are moderate, illustrating that present systems fail to retrieve events. Clearly, except for the small values of recall or precision that lead to very low F-scores, RUPEE dominates the pipeline model.

### Feature analysis

We demonstrate here the importance of the joint (trigger, theme) features in our pairwise event detector. The two main joint features are *E-walk *and *V-walk*, which represent the dependency path between text entities: as shown in Figure 4, E-walk encodes the path through the (*dep-tag, token, dep-tag*) triplet, while V-walk encodes the (*token, dep-tag, token*) triplet.

**Figure 6:**
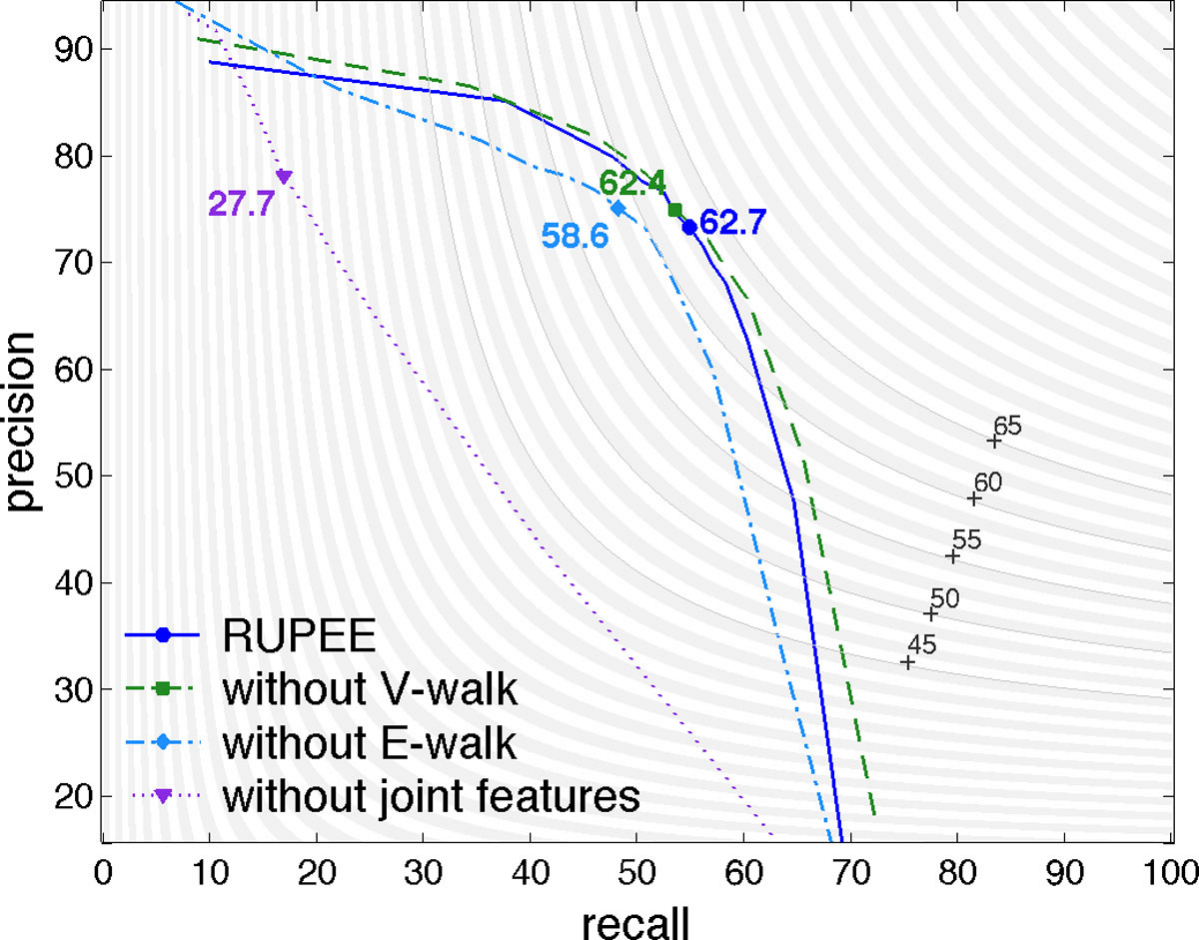
Precision recall curve of (trigger, theme) pairs classification with level curves of F-score in the background, computed on the BioNLP 2013 development set.

The importance of the joint features is measured by the loss incurred by their withdrawal from the model. The precision-recall curves are displayed in Figure 6. Without any joint features, we observe a huge drop in performance, combined with a very bad calibration of the classifier; without *E-walk*, we observe a significant drop in performance; without *V-walk*, there is a small drop, but this drop is due to bad calibration: the best calibrated classifier without *V-walk *is better than the best calibrated RUPEE classifier. Overall, the randomization approach used by [37] concludes that all pairwise differences are statistically significant at the 5% level, except for the difference between the classifier with all joint features and without *V-walk *features. Hence, joint features are important and among them, *E-walk *plays a major beneficial role, whereas *V-walk *has a marginal effect. However, we believe that *V-walk *should benefit RUPEE for larger training sets. Indeed, it is difficult to learn regularities in *V-walk *with moderate training set sizes, because the dictionary of tokens is large. As a consequence, the two tokens of the (*token, dep-tag, token*) lead to a huge feature space that is scarcely populated.

### Training durations

In this last section, we propose to illustrate the lower complexity of our approach compared to UCLEED by providing durations for training both systems on BioNLP 2011 GE. These timings do not involve preprocessing but only running cross-validation on the training set and evaluation on the development and test sets. For UCLEED, we used the code (in java & scala), which is available from [38] and we chose BioNLP 2011 GE because this code was primarily designed to run on it. Our code, in python, is publicly available from [39]. Experiments were conducted on the same computer, with a quad-core Intel Xeon CPU and 16GB of RAM. Both codes are multi-threaded and used all 4 threads simultaneously. Under these conditions, UCLEED requires around *8h30min *to run its 10 epochs, while our code completes training in about *30 min*. In addition, we also ran our pipeline counterpart system, which is implemented in the same language and uses the same libraries as RUPEE, on the same machine. The pipeline counterpart system took *20 min *to train. Note that UCLEED might be faster by using feature caching, but we had to disable it because the 16GB RAM filled up, thereby further slowing the process. In addition to the inference cost discussed earlier, the overall computation cost also depends on the training algorithms: both UCLEED and RUPEE use Support Vector Machines, with the online-learner MIRA [40] for UCLEED and a dual coordinate descent method [41] for RUPEE. We did not conduct a more thorough analysis about the training durations since the implementation choices such as programming languages and optimization details such as stopping criteria can make large differences on the computing durations.

## Conclusion

We introduced RUPEE, a recursive pairwise model designed for biomedical event extraction. RUPEE improves on the best current approaches of the BioNLP 2013 GE task. RUPEE breaks down the overall event extraction task into the classification of (trigger, theme) pairs, assigned to event types. These (trigger, theme) pairs enable to use joint features in off-the-shelf classifiers, without resorting to costly global inference models. We also implemented a recursive procedure that deals with regulation events, which may include other events in their definition. All operations are run in a unified framework, using a single event classifier.

RUPEE is fast and more accurate than the available pipeline models or joint models. Given its simplicity and scalability, we believe that RUPEE provides a strong basis for large-scale event extraction projects. Several refinements are still possible, for example by exploring other types of features, or by enabling the direct processing of triplets that may be encountered in binding or regulation events. Our experiments, which use different features of dependency paths, show that current representations of syntactic parse tree may be problematic regarding their sparsity. Continuous vector representation of pairwise relations [42] and semantic composition approaches [43] are very promising directions to generate features representing the relations between text entities. Also, bearing in mind that BioSEM [23] achieves the best performance on Binding event extraction by using complex patterns to deal with multi-argument events, going beyond pairs should be beneficial. A model that could extract higher-order relations would be expected to perform better on multi-argument event.

## Competing interests

The authors declare that they have no competing interests.

## Authors' contributions

All authors have designed the system. XL implemented it and carried out all experiments. All authors have participated in the writing, read and approved the final manuscript.

## Declarations

The publication costs of this article were funded by the French National Agency for Research through "young researchers" program (EVEREST-12-JS02-005-01).

This article has been published as part of *BMC Bioinformatics *Volume 16 Supplement 10, 2015: BioNLP Shared Task 2013: Part 1. The full contents of the supplement are available online at http://www.biomedcentral.com/bmcbioinformatics/supplements/16/S10.
